# Simulations of Gamma Cascades and Modelling Atomic Collision Chains

**DOI:** 10.6028/jres.105.015

**Published:** 2000-02-01

**Authors:** F. Bečvář, M. Krtička, M. Jentschel

**Affiliations:** Faculty of Mathematics and Physics, Charles University V Holešovičkách 2, CZ-180 00 Prague 8, Czech Republic; Institut Laue-Langevin, F-38042 Grenoble, France

**Keywords:** atomic collisions, Doppler broadening, gamma cascades, gamma spectra, heavy nuclei, lifetimes, neutron capture, nuclear levels, Monte Carlo simulation

## Abstract

A new method for simulation of nuclear γ cascades by the Monte Carlo technique is described. It makes it possible to generate artificially individual events of the γ-cascade decay of neutron capturing states in heavy nuclei. For the purpose of determination of lifetimes of nuclear levels by the GRID method the previously developed Mean Free Path Approach for modelling atomic collisions and their interplay with cascades has been generalized. Examples of the use of this generalization for determination of lifetimes of selected ^150^Sm and ^158^Gd levels are given and discussed.

## 1. Introduction

The GRID method [1] for the determination of lifetimes of levels in heavy nuclei is applicable provided that there exists a precise enough way of predicting the distribution for the velocity of the deexciting nucleus at the moment when the level of interest is being depopulated. In fact, this information is needed separately for various assumed values of the lifetime of the level studied to predict corresponding Doppler γ-line profiles. These profiles are compared with the experimentally measured profile and the lifetime can in turn be estimated.

In the case of slow-neutron capture in heavy nuclei the actual nuclear velocity distribution is shaped by time-distributed nuclear recoils, originating from emissions of a broad variety of γ cascades, and by the collisions of the atom, containing the deexciting nucleus, with its environment. While γ cascades, whose behaviour is largely statistical, are a source of motion for the nucleus, the collisions with surrounding atoms tend to slow-down this motion.

It seems that the only possibility of getting the required velocity distributions for heavy nuclei is provided by modelling these two competing processes and their interplay with the aid of the Monte Carlo technique.

The global scheme we adopted consists in a simulation of data about a large number of γ cascades and subsequent modelling the above-outlined interplay in which these data are repeatedly used under various assumptions about the lifetime, as already outlined.

The present paper describes briefly the method for simulating γ cascades based on the use of the algorithm DICEBOX [2] and demonstrates its applicability. The previously developed Mean Free Path Approach (MFPA) for modelling atomic collisions and their interplay with cascades, see Ref. [3], has been generalized. This generalization, referred to as the Fluctuating Free Path Approach (FFPA), has been used for estimating lifetimes of selected levels in ^150^Sm and ^158^Gd. The results obtained are discussed.

## 2. Simulation of γ Cascades

### 2.1 Algorithm

The algorithm DICEBOX [2] is based on the validity of the extreme statistical model of a nucleus and further simplifying assumptions as follows:

1. Below a certain critical energy *E*_crit_ a full set of levels is known, including level energies *E*, spins *J*, parities π and all branching intensities.
2. A full, presumably unknown set of level energies of a nucleus above *E*_crit_ represents a random discretization of an *a priori* known level density formula *ρ*(E, J^π^).
3. In the case of a transition of pure type X (X = E, M) and multipolarity *L* the corresponding partial radiative width *Γ_aγb_*, governing the γ decay of a level *a* with energy above *E*_crit_ to a level *b*, is assumed to be a random choice from the Porter-Thomas distribution whose mean value langle 〈*Γ_aγb_*〉 is uniquely determined by the level density *ρ*(E, J^π^) and the corresponding, *a priori* known photon strength function $S_{\gamma }^{\left(XL\right)}(E_{\gamma })$, where *E*_γ_ is the γ-ray energy.

Regarding assumptions (2) and (3), it should be stressed that existing data on level densities and photon strengths for heavy nuclei are very limited, especially at energies of around 3 MeV. Several theoretical models for these quantities have been developed, but in view of the deficiency of experimental data in many instances it is very difficult to decide which of these models are closer to reality.

In the case of a typical heavy nucleus, the total number of levels below the neutron capturing state reaches the value as high as ≃3×10^6^. As a consequence, the total number of partial widths *Γ_aγb_* is enormous, up to 10^13^. Hereafter, for these levels and all these widths we use the term *nuclear realization.* In accordance with assumptions (2) and (3) there exist an infinite number of nuclear realizations, one of them being represented by the *actual* set of levels and partial widths.

In our approach the simulation of γ cascades consists of two stages: (i) the artificial construction of a complete decay scheme, embodied by a random choice of a nuclear realization; (ii) the proper simulation of the γ cascades governed by this scheme that yields energies of the individual γ rays and their multipolarities, as well as energies and total radiation widths of the encountered intermediate levels.

The myriads of partial radiation widths display violent Porter-Thomas fluctuations. As a consequence, if various nuclear realizations are chosen in the process of simulations of γ cascades, modelling the interplay between the atomic collisions and the γ emissions will lead in principle to different predictions of nuclear velocity distributions and consequently to different lifetime estimates. In other words, even if the level density formula *ρ* (E, J^π^) and all photon strength functions $S_{\gamma }^{\left(XL\right)}\left(E_{\gamma }\right)$ together with their parameters were precisely known, there would be still some residual uncertainty in lifetime determination. To assess this uncertainty it is important to model velocity distributions and resulting γ-line profiles for a reasonably large set of randomly selected nuclear realizations.

The simulation of γ-cascades involves the above-mentioned discretization of level-density formula *ρ* (E, J^π^) together with the randomization of partial radiation widths *Γ_aγb_*. In our algorithm this is achieved with the aid of usual procedures based on the Monte Carlo technique. When discretizing the level density, the Wigner repulsion between energies of neighbouring levels of the same spin and parity is taken into account. The process of getting a nuclear realization implicitly involves the use of a deterministic generator of uniformly distributed random numbers whose operation is controlled by a parameter known as the *generator seed*.

**Fig. 1:**
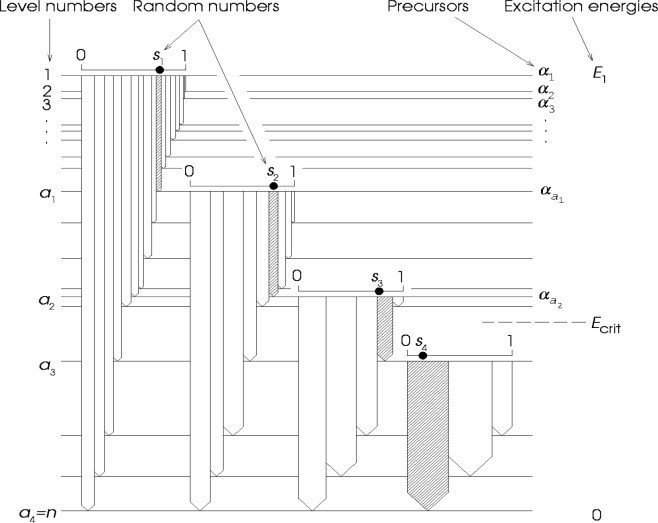
Schematic description of random cascading.

Assume for a while that realizations of all, say, 10^13^ partial radiation widths *Γ_aγb_*. are at our disposal and so also all branching intensities *I_aγb_*, characterizing depopulation of any level *a* below neutron binding energy to individual levels *b*. With these data it would be easy to simulate event by event the emissions of individual γ cascades, as well as the lifetimes of the intermediate levels involved. However, in view of the enormous number of required widths, this assumption is evidently unrealistic. In order to overcome this difficulty the algorithm DICEBOX provides random realizations of partial radiation widths *Γ_aγb_* only for restricted pairs of levels [*a,b*]. Specifically, these realizations are being obtained step by step only for all those possible pairs [*a,b*] for which their first member *a* equals to *a*_0_, *a*_1_, *a*_2_, …, where *a*_0_ is the neutron capturing state and *a*_1_, *a*_2_, … are the intermediate levels encountered consecutively during deexcitation by a current γ cascade. The process of obtaining restricted sets of partial radiation widths is depicted in Fig. 1, where widths for sets of pairs [*a_i_*, *b*] are represented by corresponding sets of branching intensities. Figure 1 also illustrates the way how the uniformly distributed random numbers, *s_i_*, are used for the selection of the individual steps of the four-step cascade shown; these steps are represented by hatched arrows.

In keeping with the introduced concept of nuclear realizations it is important to guarantee that each time when a full set of partial radiation widths for a given level *a* is needed, the above process yields the *same* set of values *Γ_aγb_*. As explained in detail in Ref. [2], this requirement is satisfied by the introduction of so-called precursors. These are represented by random generator seeds *α_a_*, randomly ascribed to all levels *a* with energies *E_a_* in between the critical energy *E*_crit_ and the energy of neutron capturing state *E*_1_, see Fig. 1. Whenever a full set of widths *Γ_aγb_* for a fixed *a* is to be generated, the random generator used is preset with the aid of the corresponding precursor *α_a_*.

### 2.2 Examples

The described method for the simulation of γ cascades has been applied to analyses of data from measurements of Two-Step Cascades (TSCs) following the capture of thermal neutrons. From these analyses validity of the existing models for photon strength functions were tested at intermediate energy region near 3 MeV. Conclusions reached for ^146^Nd and ^163^Dy are given in Refs. [4, 5]. The same method has been used as a quantitative basis for spin and parity determination of neutron *s*- and *p*-wave resonances of ^113^Cd and ^107^Ag targets, see Refs. [6, 7]. Also in these cases, conclusions about photon strengths could be drawn.

**Fig. 2:**
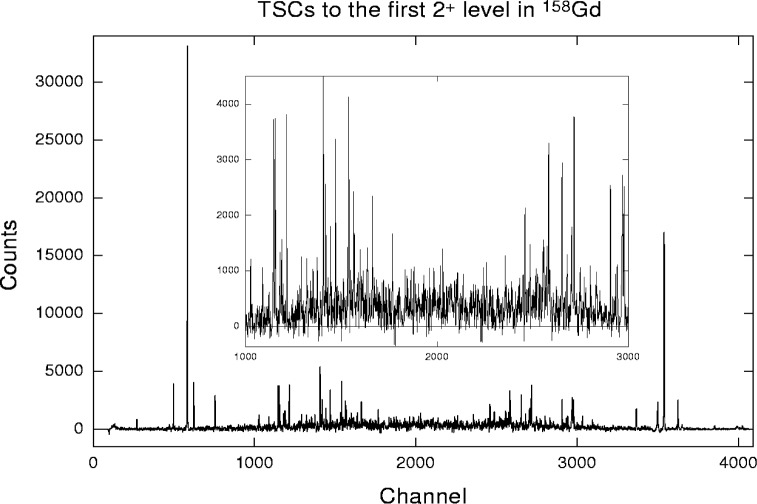
Spectrum of TSCs terminating at the first J^π^ = 2^+^ level in ^158^Gd.

**Fig. 3:**
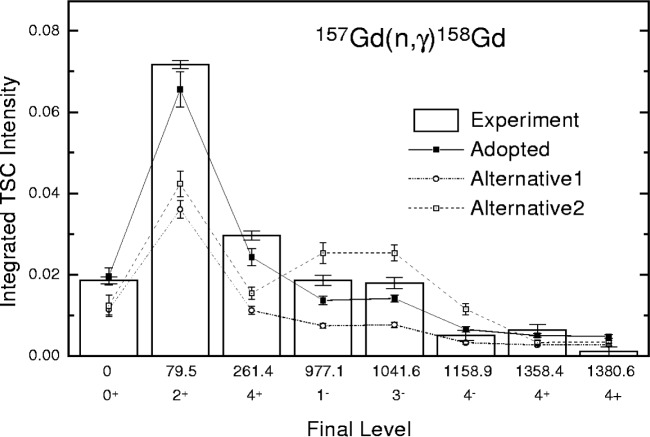
Experimental and modelled integrated intensities of TSCs terminating at various low-energy levels in ^158^Gd.

An additional example is the analysis of the unpublished data on TSCs, following the thermal neutron capture in ^157^Gd. This reaction was studied at the 15 MW research reactor at Řež near Prague using the method, described in Ref. [8]. An example of a TSC γ-ray spectrum is given in Fig. 2. The *integrated* TSC intensities that belong to various levels in ^158^Gd at which TSCs terminate are plotted in Fig. 3. These intensities were obtained by the integration of the corresponding TSC spectra over the 3 MeV interval centered around the midpoints of the spectra.

Modelling of TSCs was undertaken for each of three choices of photon strength functions and the level-density formula, as specified in Table 1. All of these choices led to the values of total radiation width of the neutron capturing state that are in reasonable agreement with the known experimental value *Γ*_γ_ = 97±8 meV.

The integrated TSC intensities deduced from this modelling are plotted in Fig. 3; the attached error bars represent the fluctuations due to inherent statistical character of nuclear realizations.

It is evident that the option referred to as “Adopted” leads to an acceptable reproduction of the experimentally measured integrated TSCs intensities, while the other two considered alternatives seem to be ruled out. This suggests that the traditional model of Axel and Brink [11, 12] for the E1 strength is not acceptable, while the validity of the model of Kadmenskij, Markushev, and Furman [13], seems to have been supported. Note that the model of Axel and Brink assumes the energy-independent damping width of the giant electric dipole resonance, while the model of Kadmenskij et al. introduces the energy and temperature dependence. The disagreement with the model of Axel and Brink is surprising, as this model leads to reasonable predictions of the E1 photon strength for deformed rare-earth nuclei at energies of primary γ rays, i.e., at 6 to 8 MeV. Nevertheless, as shown in Ref. [14] for the case of the neighbour ^157^Gd compound nucleus, the model of Kadmenskij et al. may still play some role. With this, it should be stressed that this model represents only a low-energy approximation. It may be the case that the true E1 photon strength function is close to such an approximation at low energies, while at 6 to 8 MeV it tends towards the predictions of the model of Axel and Brink. The comparison in Fig. 3 suggests that the M1 photon strength comes from simultaneous contributions of the scissors resonance and the giant magnetic dipole resonance which are assumed to be built upon *each level* in ^158^Gd, supporting the analogous conclusion for the ^163^Dy compound nucleus [5].

Notwithstanding the progress in understanding the behaviour of photon strengths during the last decade, the discussed analysis of TSCs in ^158^Gd indicates still persisting problems. To be strict, there exists also a problem of correct choice of level density formula, which represents the second potential hurdle in rigorous simulations of γ cascades.

## 3. Fluctuating Free Path Approach

The application of the previously developed MFPA [3] leads to values of lifetime estimates that are at average higher by a factor of 1.35, compared to estimates, yielded by other, presumably more reliable methods, see Ref. [15]. Here we mean the well-established DSAM method and—in case of simple feeding patterns—also the GRID method combined with true Molecular Dynamic (MD) modelling. The difficulties with biased estimates of lifetime motivated us to develop the FFPA as a sophistication of the MFPA.

### 3.1 Brief Description

The successive emissions of γ rays in a cascade are assumed not to be angularly correlated and the time interval between any pair of them is treated as a quantity randomly drawn from the exponential distribution whose “lifetime parameter” is deduced from the total radiation width of the appropriate intermediate level. The recoil velocity *v* is then given by a simple expression

$$v=\frac{E_{\gamma }}{mc},$$ (1)

where *m* is a mass of the colliding atom and *E*_γ_ is the energy of the γ ray inducing the recoil.

Only binary, classical hard-sphere collisions between the projectile atom and atoms of the sample are considered. Similarly, as in the MFPA, the energy-dependent hard-sphere radius is deduced from equality of the initial kinetic energy *ϵ*_in_ in the center-of-mass system to the Born-Mayer potential. In this system the angular distribution of atoms is understood to be isotropic, which implies that energy losses are variable. The velocities of atoms of the sample follow the Maxwell-Boltzmann distribution. As in MFPA, no corrections for inaccessible volume inside the assumed hard spheres are performed. Actual free paths of colliding atoms are fluctuating around their average for a given energy *ϵ*_in_. While calculating the cross sections for collision of projectile and target atoms, the “velocity aberration,” represented by factor |***V*** – ***v***|/| ***v***|, is taken into account. This cross section is

$$\sigma =\frac{\left|V-v\right|}{\left|v\right|}{\left\{a\;\ln\left[\frac{2\left(M+m\right)A}{mM{\left|V-v\right|}^{2}}\right]\right\}}^{2},$$ (2)

where *M* is the mass of the target, while ***v*** and ***V*** are velocity vectors of the target and projectile, respectively; quantities *a* and *A* are parameters of the Born-Mayer potential.

### 3.2 Testing the Approach

In order to make comparison of the FFPA to a presumably more reliable approach, we modelled evolution of velocity distribution for colliding atoms using the FFPA and the Restricted Molecular Dynamic (RMD) approach. With both the mentioned above approaches we modelled collisions of Sm atoms in a sample of Sm_2_O_3_, assuming that the only source of movement of colliding atoms is the initial recoil, represented by a fixed velocity of 0.15 Å fs^−1^. Such a velocity represents approximately a recoil from a typical primary capture γ ray.

In our RMD modelling we assumed a finite cell of fixed volume formed by *N* = 3200 atoms of the sample with no interaction between any pair of them. The additional atom enters as a projectile with the above-mentioned fixed velocity and interacts with each of the *N* atoms, the interaction being governed by the Born-Mayer repulsive potential. Atoms of the cell start to move. Trajectories of all *N* + 1 atoms are obtained from solution of classical equations of motion. A periodic boundary condition is imposed. Thus, if some of *N* + 1 atoms leaves the volume of the cell, it will reappear on the “opposite side” of the cell preserving its velocity.

**Fig. 4:**
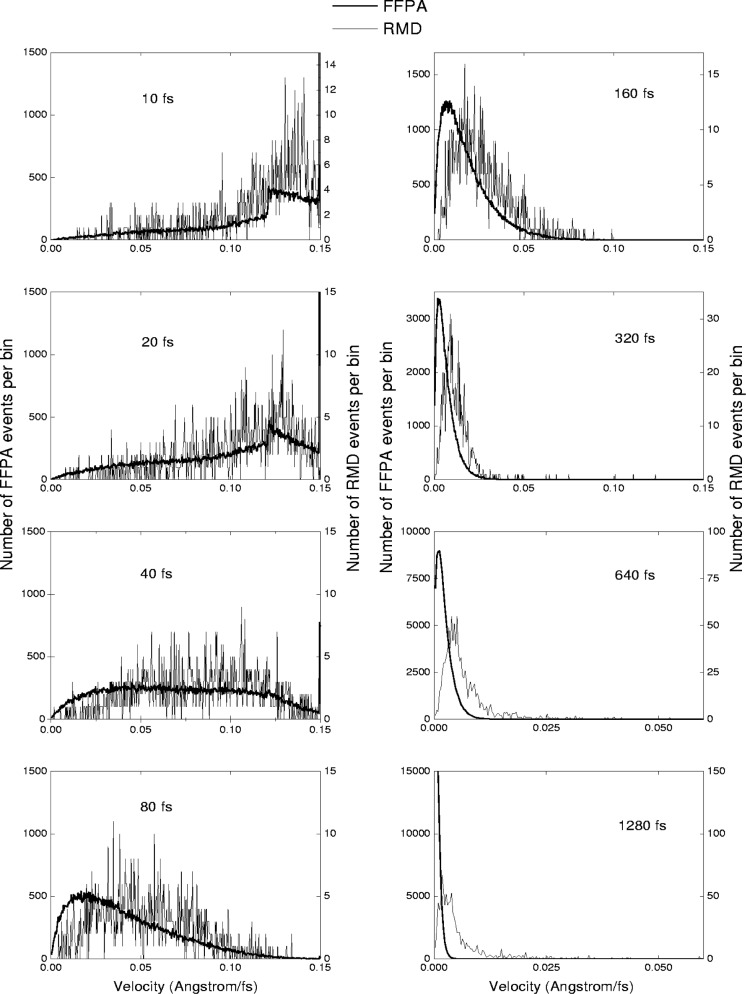
Comparisons between distributions of velocities of Sm atoms obtained from the FFPA and RMD modelling at various times of slowing down from (10 fs to 1280 fs). Initial velocities of Sm atoms were adjusted to 0.15 Å fs^−1^.

The FFPA and RMD predictions of velocity distributions are illustrated in Fig. 4. It can be seen that for short times of atomic slowing down the velocity distributions obtained are very close to each other, which seems to be the most important conclusion from this comparison, suggesting that our strongly simplified modelling atomic collisions at early stages of this process is correct.

The apparent disagreement for times of slowing down exceeding 100 fs can be, at least in part, accounted for by the following three mutually related artifacts of RMD modelling: (i) the formation of a thermal spike followed by its subsequent fading away, (ii) the gradual warming-up of a relatively small cell due to the energy deposited from the projectile, and (iii) the thermalisation of the projectile. To test the correctness of the FFPA at the full time scale, more sophisticated MD modelling is to be performed yet.

## 4. Examples of Lifetime Estimates and Discussion

**Fig. 5:**
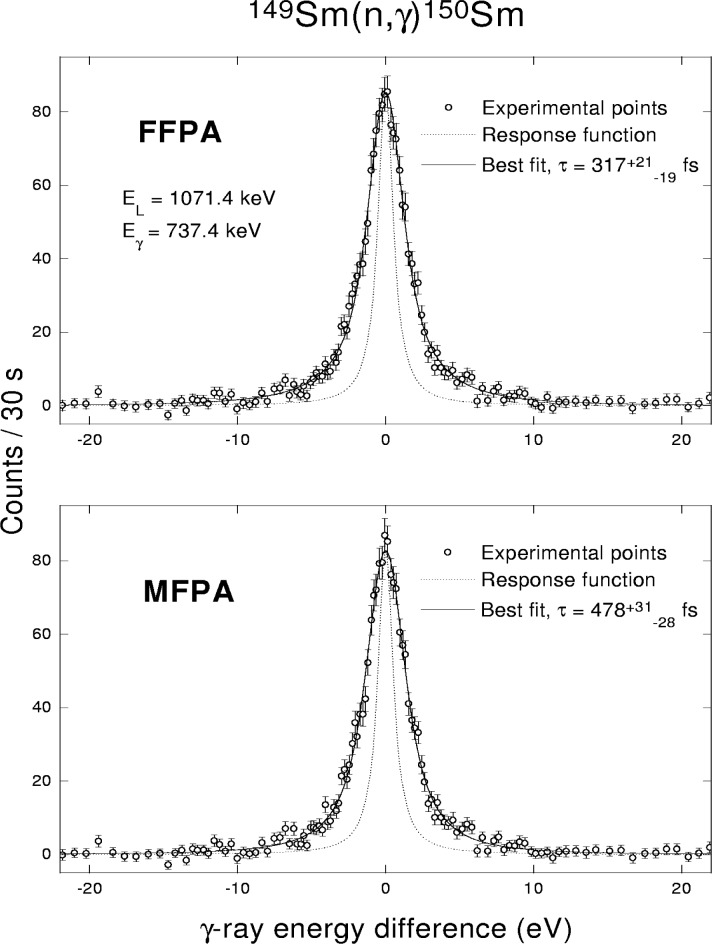
The profile of the 737.4 keV line, resulting from depopulation of the J^π^ = 3^+^ level at 1071.4 keV in ^150^Sm, fitted within the FFPA and MFPA.

A case of routine application of the FFPA in conjunction with the simulation of γ cascades by the DICEBOX algorithm, described in Sec. 2.1, is illustrated in Fig. 5. The shown profile of the 737.4 keV γ line determined from GRID (n,γ) measurements with ^149^Sm target is satisfactorily reproduced and the lifetime for the corresponding level of interest in ^150^Sm at 1071.4 keV is estimated to be $318_{-21}^{+25}$ fs.

For comparison, results obtained with the aid of the MFPA are also shown in Fig. 5. As it is evident, both the approaches, FFPA and MFPA, lead to indistinguishable best fits of the experimental line profile. However, the determination of lifetime within the MFPA yields a value $478_{-28}^{+31}$ fs which differs significantly from what has been obtained using the FFPA.

The same conclusion could be drawn from analyses of 16 γ-line profiles deduced from GRID measurements with ^157^Gd target [16]. In this case it has been found that the ratio between lifetime estimates, *τ*_FFPA_/*τ*_MFPA_, varies within the interval from 0.65 to 0.8, displaying a smooth dependence on lifetime in the region from 30 ps to 5000 ps. Although there is no direct comparison between the FFPA and the approaches based on the DSAM or the true MD modelling, it seems that FFPA estimates are biased in a substantially lower degree than those provided by the MFPA.

In Table 2 the FFPA lifetime estimates of selected ^158^Gd levels are listed for all three choices of $S_{\gamma }^{\left(XL\right)}$(*E*_γ_) and *ρ*(E,J^π^) specified in Table 1. It is evident that for long-lived levels all of these choices lead virtually to the same lifetime estimates. In contrast, for short-lived levels the lifetimes deduced vary strongly from choice to choice.

**Fig. 6:**
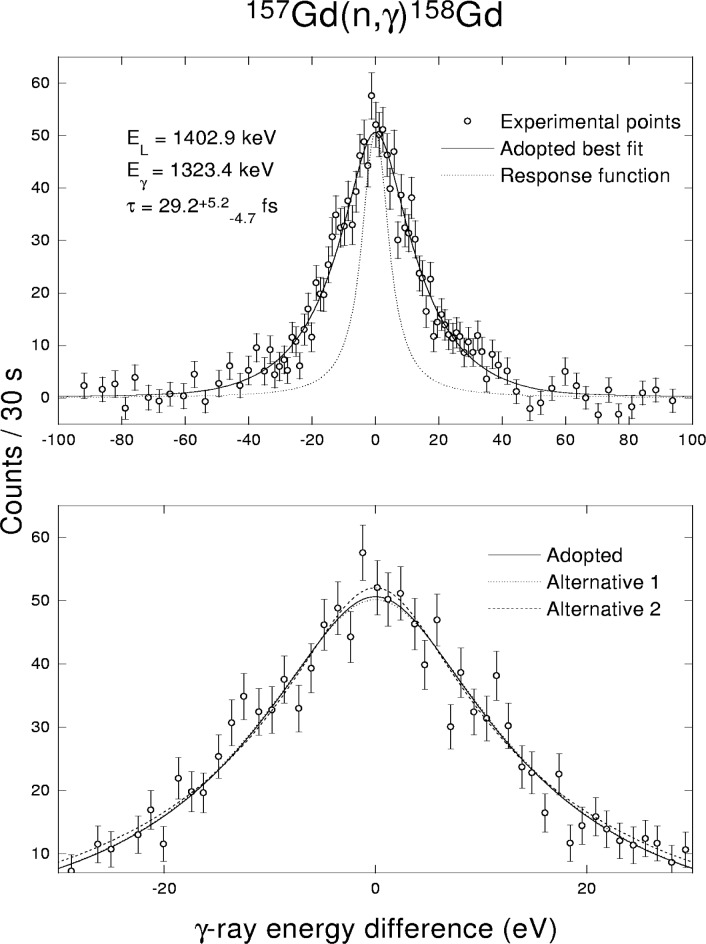
The profile of the 1323.4 keV line, resulting from depopulation of the 1402.9 keV level in ^158^Gd, fitted within the FFPA. Three different choices for photon strength functions and the level-density formula were used, as specified in Table 1.

Regarding the short-lived ^158^Gd levels, the modelled line profiles for the individual choices of $S_{\gamma }^{\left(XL\right)}$(E_γ_) and *ρ*(E,J^π^) turned out to be almost the same, see the example in Fig. 6. The shape of experimentally determined line profile itself could not thus set a meaningful constraint on the choice of the entities discussed. On the other hand, as is evident from the discussion in Sec. 2.2, the data on TSCs for ^158^Gd yielded some limitation for the choice of photon strength functions. As data in Table 2 indicate, for correct determination of short lifetimes by the GRID technique the strength functions must have very finely tuned shapes and sizes. Unfortunately, because of lack of adequate experimental data, this is not the case for majority of heavy nuclei.

As already pointed out in Sec. 2.1, the inherent statistical behaviour of nuclear realizations is a source of residual uncertainties in determination of lifetimes. Table 3 lists such uncertainties for three selected levels in ^158^Gd and compare them with the experimental uncertainties, associated with finite precision of measurements of γ-line profiles. The data in Table 3 suggest that residual uncertainties may limit accuracy of lifetime determination only in cases of extremely short lifetimes. It is worthwhile to note that the role of such uncertainties is expected to be enhanced in cases of even-even product nuclei that display low level densities.

## 5. Concluding Remarks

We stress that our proposed FFPA is still based on a very simplified scenario of interatomic collisions. Nevertheless, this approach seems to work, reducing the long-lasting problem with overestimating lifetimes by the MFPA.

Regarding simulations of γ cascades, the still open questions concerning quantities $S_{\gamma }^{\left(XL\right)}$ (*E*_γ_) and *ρ*(E,J^π^) represent the only serious limitation. In view of this difficulty the *routine use* of the FFPA for estimating lifetimes of *short-lived* levels (*τ*<100 ps) in *heavy nuclei* by the GRID method becomes problematic.

The need for data from dedicated experiments is clearly indicated. Of special importance seem the conventional (n,γ) measurements at isolated *s*- and *p*-resonances, the studies of TSCs following the capture of thermal neutrons and, in particular, future possible studies of multistep γ cascades with the aid of sophisticated technique of the BaF_2_ or HPGe crystal balls. Regarding multistep cascades it would be interesting to perform the measurements at neutron energies of several tens of keV when contributions of *p*-wave neutrons become important. All these studies seem to be crucial not only for better determination of photon strength functions in specific cases, but mainly for understanding systematic behaviour of photon strength functions and the level-densities in a broad class of heavy nuclei.

On the other hand, the limited knowledge of photon strength functions does not seem to be a snag in determination of long lifetimes. However, in order to avoid a bias in lifetime estimates in such cases, thoroughgoing tests of the FFPA are to be undertaken yet.

## Figures and Tables

**Table 1:** Photon strength functions and level-density formulas used in modelling the TSCs, following the ^157^Gd(n,γ)^158^Gd reaction

Choice	Model for photon strength			Level densiy	*Γ*_γ_ (meV)
	E1	M1	E2		
Adopted	KMF^a^	GDR+SR^b^	SP^c^	BSFG^d^	92
Alternative 1	KMF	SP	SP	BSFG	108
Alternative 2	AB^e^	GDR+SR	SP	CTF^f^	114

a The model of Kadmenskij, Markushev and Furman [13].

b The model assuming the existence of the giant dipole and scissors magnetic resonances, see e.g. Ref. [5].

c The single-particle model assuming energy-independent $S_{\gamma }^{\left(XL\right)}$ (*E*_γ_) [9].

d The level-density formula according to the back-shifted Fermi-gas model, see e.g. Ref. [10].

e The model of Axel and Brink [11,12].

f The constant-temperature level-density formula, see e.g. Ref. [10].

**Table 2:** Estimates of lifetimes for levels in ^158^Gd obtained for three different choices of the photon strength functions and the level density formula. The choices, referred to as “Adopted,” “Alternative 1” and “Alternative 2,” are specified in Table 1

Level energy (keV)	Transition energy (keV)	Lifetime (fs)		
		Adopted	Alternative 1	Alternative 2
977.1	897.6	$938_{-135}^{+181}$	$918_{-130}^{+170}$	$947_{-131}^{+172}$
1176.4	915.0	$286_{-27}^{+31}$	$268_{-25}^{+29}$	$276_{-27}^{+31}$
1196.1	1116.5	$3867_{-1056}^{+2288}$	$3975_{-752}^{+1126}$	$3803_{-741}^{+1095}$
1263.5	1263.5	$39.5_{-6.8}^{+7.5}$	$31.5_{-6.5}^{+7.2}$	$54.8_{-7.0}^{+7.9}$
1402.9	1141.4	$36_{-4.7}^{+5.0}$	$20.7_{-4.6}^{+4.8}$	$49.6_{-4.9}^{+5.2}$
1402.9	1323.4	$29.2_{-4.7}^{+5.2}$	$10.7_{-4.2}^{+4.8}$	$48.9_{-5.2}^{+5.5}$

**Table 3:** Experimental and residual uncertainties in estimating lifetimes of selected levels in ^158^Gd, as obtained within the FFPA

Level energy (keV)	Transition energy (keV)	*τ* (fs)	Uncertainty of *τ* (fs)	
			Residual	Experimental
1263.5	1184.0	42.1	±4.1	+4.1, −3.8
977.1	897.6	938	±18	+181, −136
1158.9	897.6	2535	±219	+863, −469
